# Concomitant Evaluation of Heart Period and QT Interval Variability Spectral Markers to Typify Cardiac Control in Humans and Rats

**DOI:** 10.3389/fphys.2019.01478

**Published:** 2019-11-29

**Authors:** Beatrice De Maria, Vlasta Bari, Andrea Sgoifo, Luca Carnevali, Beatrice Cairo, Emanuele Vaini, Aparecida Maria Catai, Anielle Cristhine de Medeiros Takahashi, Laura Adelaide Dalla Vecchia, Alberto Porta

**Affiliations:** ^1^IRCCS Istituti Clinici Scientifici Maugeri, Milan, Italy; ^2^Department of Cardiothoracic, Vascular Anesthesia and Intensive Care, IRCCS Policlinico San Donato, Milan, Italy; ^3^Stress Physiology Laboratory, Department of Chemistry, Life Sciences and Environmental Sustainability, University of Parma, Parma, Italy; ^4^Microbiome Research Hub, University of Parma, Parma, Italy; ^5^Department of Biomedical Sciences for Health, University of Milan, Milan, Italy; ^6^Department of Physiotherapy, Federal University of São Carlos, São Carlos, Brazil

**Keywords:** power spectral analysis, heart rate variability, QTV, ventricular repolarization, autonomic nervous system, wild-type rat, Wistar, head-up tilt

## Abstract

The variability of heart period, measured as the time distance between two consecutive QRS complexes from the electrocardiogram (RR), was exploited to infer cardiac vagal control, while the variability of the duration of the electrical activity of the heart, measured as the time interval from Q-wave onset to T-wave end (QT), was proposed as an indirect index of cardiac sympathetic modulation. This study tests the utility of the concomitant evaluation of RR variability (RRV) and QT variability (QTV) markers in typifying cardiac autonomic control of humans under different experimental conditions and of rat groups featuring documented differences in resting sympatho-vagal balance. We considered: (i) 23 healthy young subjects in resting supine position (REST) undergoing head-up tilt at 45° (T45) and 90° (T90) followed by recovery to the supine position; (ii) 9 Wistar (WI) and 14 wild-type Groningen (WT) rats in unstressed conditions, where the WT animals were classified as non-aggressive (non-AGG, *n* = 9) and aggressive (AGG, *n* = 5) according to the resident intruder test. In humans, spectral analysis of RRV and QTV was performed over a single stationary sequence of 250 consecutive values. In rats, spectral analysis was iterated over 10-min recordings with a frame length of 250 beats with 80% overlap and the median of the distribution of the spectral markers was extracted. Over RRV and QTV we computed the power in the low frequency (LF, from 0.04 to 0.15 Hz in humans and from 0.2 to 0.75 Hz in rats) band (LF_RR_ and LF_QT_) and the power in the high frequency (HF, from 0.15 to 0.5 Hz in humans and from 0.75 to 2.5 Hz in rats) band (HF_RR_ and HF_QT_). In humans the HF_RR_ power was lower during T90 and higher during recovery compared to REST, while the LF_QT_ power was higher during T90. In rats the HF_RR_ power was lower in WT rats compared to WI rats and the LF_QT_ power was higher in AGG than in non-AGG animals. We concluded that RRV and QTV provide complementary information in describing the functioning of vagal and sympathetic limbs of the autonomic nervous system in humans and rats.

## Introduction

Heart period, measured as the time distance between two consecutive QRS complexes from the electrocardiogram (RR), exhibits spontaneous fluctuations usually referred to as RR variability (RRV). The analysis of RRV provides some markers that have been found useful to infer the state of the cardiac autonomic control (Task Force of the European Society of Cardiology and the North American Society of Pacing and Electrophysiology,, 1996). Short-term RRV markers in humans are mainly associated with vagal modulation given that the magnitude of RR changes is dramatically reduced by full vagal blockade (Pomeranz et al., 1985). This consideration holds not only in humans but also in rats (Japundzic et al., 1990; Cerutti et al., 1991; Silva et al., 2017) and this analogy strengthened the use of rats as an animal model of human autonomic cardiac control. The amplitude of the respiratory sinus arrhythmia is one of the most utilized RRV indexes to typify cardiac vagal control (Hirsch and Bishop, 1981): it is frequently estimated via spectral analysis as the power of RRV in the high frequency (HF) band in both humans and rats, even though the definition of the HF band was adapted to account for the differences between the respiratory rates in the two species, namely from 0.15 to 0.5 Hz in humans and from 0.75 to 2.5 Hz in rats (Japundzic et al., 1990; Cerutti et al., 1991; Rubini et al., 1993). In particular, in humans the HF power of RRV is known to decrease during physiological conditions characterized by sympathetic activation and vagal withdrawal, such as during graded orthostatic challenge (Montano et al., 1994; Cooke et al., 1999; Porta et al., 2011; Marchi et al., 2016) or physical exercise (Shin et al., 1995a, b; Brenner et al., 1997; Porta et al., 2018). Similarly, in rats the HF power of RRV was utilized to typify the autonomic response to several types of stressors either pharmacological, interventional, or social (Akselrod et al., 1987; Japundzic et al., 1990; Cerutti et al., 1991; Rubini et al., 1993; Stauss et al., 1997; Sgoifo et al., 1998, 1999; Jaenisch et al., 2011; Carnevali et al., 2013; Carnevali and Sgoifo, 2014; Silva et al., 2016, 2017).

More recently, in parallel with the more traditional RRV analysis, the variability of the overall duration of the electrical activity of the heart, comprising depolarization and repolarization periods, usually quantified as the time interval from Q-wave onset to T-wave end (QT) from the electrocardiogram, has been proposed and validated as a marker of cardiac sympathetic control in humans (Berger, 2009; Malik, 2009; Porta et al., 2010; Baumert et al., 2016). QT variability (QTV) markers computed in the low frequency (LF) band (i.e., from 0.04 to 0.15 Hz in humans) have been found to increase in situations where sympatho-vagal balance is shifted toward sympathetic activation and vagal withdrawal, especially when the sympathetic drive is particularly high (Lombardi et al., 1996; Porta et al., 1998a, 2010, 2011; Yeragani et al., 2000a, b; Piccirillo et al., 2001, 2006; Bar et al., 2007; Baumert et al., 2008, 2011; El-Hamad et al., 2015), with relevant clinical consequences in risk stratification (Berger et al., 1997; Atiga et al., 1998; Porta et al., 2015). Conversely, no information was provided about the possibility to use QTV in rats, mainly because of the technical difficulties in reliably assessing QT fluctuations due to the very limited signal-to-noise ratio of QTV (Laguna et al., 1990; Speranza et al., 1993; Lombardi et al., 1996; Porta et al., 1998b) and the peculiarities of cardiac repolarization in rodents (Conrath and Opthof, 2006; Fabritz et al., 2010; Speerschneider and Thomsen, 2013; Boukens et al., 2014).

The aim of the present study is to propose the concomitant evaluation of RRV and QTV to provide a more complete view on cardiac autonomic control and to test whether this strategy could be fruitfully exploited in both humans and rats. The hypothesis of the study is that the concomitant evaluation of RRV and QTV markers can describe simultaneously cardiac vagal control via the analysis of the RRV and cardiac sympathetic regulation via the analysis of the QTV in both humans and rats. In humans we evaluated two situations of sympathetic activation and vagal withdrawal of different intensities, namely head-up tilt at 45° and 90° (Montano et al., 1994), and the following recovery to supine position during which a progressive decline of sympathetic control and a gradual vagal rebound are expected. In rats, we considered two strains with documented differences in resting cardiac sympatho-vagal balance, namely the Wistar (WI) and wild-type Groningen (WT) rats (Carnevali and Sgoifo, 2014), and, within the WT population, two subgroups featuring opposite levels of aggressiveness that have been linked to different states of the cardiac autonomic control (Carnevali et al., 2013).

## Materials and Methods

### Experimental Protocol on Humans

We studied 23 young healthy volunteers (11 males, age: 26.3 ± 5.6 years). A detailed medical history and examination excluded the evidence of any disease. The subjects did not take any medication and consume any caffeine or alcohol-containing beverages in the 24 h before the recording session. Each subject underwent two consecutive head-up tilt tests with different table inclination angles, namely 45° (T45) and 90° (T90). T45 and T90 sessions were carried out in a random order, lasted 10 min and were followed by 40 min of recovery (R45 and R90, respectively) starting when the tilt table was moved back to the horizontal position. The first tilt session was preceded by a 10-min recording period in supine position (REST). Subjects lay on the tilt table supported by two belts at the level of the thigh and waist, respectively, and with both feet touching the footrest of the tilt table. During the recording sessions, subjects breathed spontaneously but were not allowed to talk. The electrocardiographic activity from a modified lead II was recorded (Biosignal Conditioning Device, Marazza, Monza, Italy) throughout all the experimental sessions and sampled at 1000 Hz. Attention was paid during the positioning of the electrodes to prevent flat or biphasic T-waves. All subjects were able to complete the protocol without experiencing any sign of presyncope. The duration of the phases was never varied.

Informed consent was obtained from all subjects before taking part in the study. The study adheres to the principles of the Declaration of Helsinki for medical research involving human subjects. The Human Research and Ethical Review Board of the L. Sacco Hospital, Milan, Italy, approved the protocol.

### Experimental Protocol on Rats

We studied two different strains of rats: 9 male WI rats (age: 5.5 ± 0.5 months; weight: 436 ± 34 g) and 14 male WT rats (age: 4.4 ± 0.5 months; weight: 395 ± 40 g). Initially WT rats were classified into non-aggressive (non-AGG, *n* = 5) and aggressive (AGG, *n* = 9) WT rats, according to the resident intruder test described in Carnevali et al. (2013).

After the preliminary behavioral tests in WT rats, all rats were implanted, under tiletamine hydrochloride plus zolazepam hydrochloride anesthesia (Zoletil Virbac, France, 20 mg kg^–1^), with radiotelemetric transmitters (TA11CTA-F40, Data Sciences International, St. Paul, MN, United States) for the recording of the cardiac electrical activity. Electrocardiograms were picked up by platform receivers (RPC-1, Data Sciences Int., St. Paul, MN, United States) located under the animal’s cage and acquired via ART-Gold 4.2 data acquisition system (Data Sciences International, St. Paul, MN, United States) at a sampling rate of 1000 Hz. Animals were individually housed and kept in rooms with controlled temperature (22 ± 2°C) and lighting (lights on from 7:00 P.M. to 7:00 A.M.). After a 14-day recovery period from surgery, electrocardiograms were recorded in all rats for 1 h during the dark (active) phase of the light–dark cycle (i.e., between 10:00 A.M. and 11:00 A.M.) on different days.

The experimental protocol was approved by the Veterinarian Animal Care and Use Committee of the University of Parma, Parma, Italy, and the animals were cared in accordance with the European Community Council Directives of 22 September 2010 (2010/63/UE).

### Extraction of the Beat-to-Beat RRV and QTV Series

**FIGURE 1:**
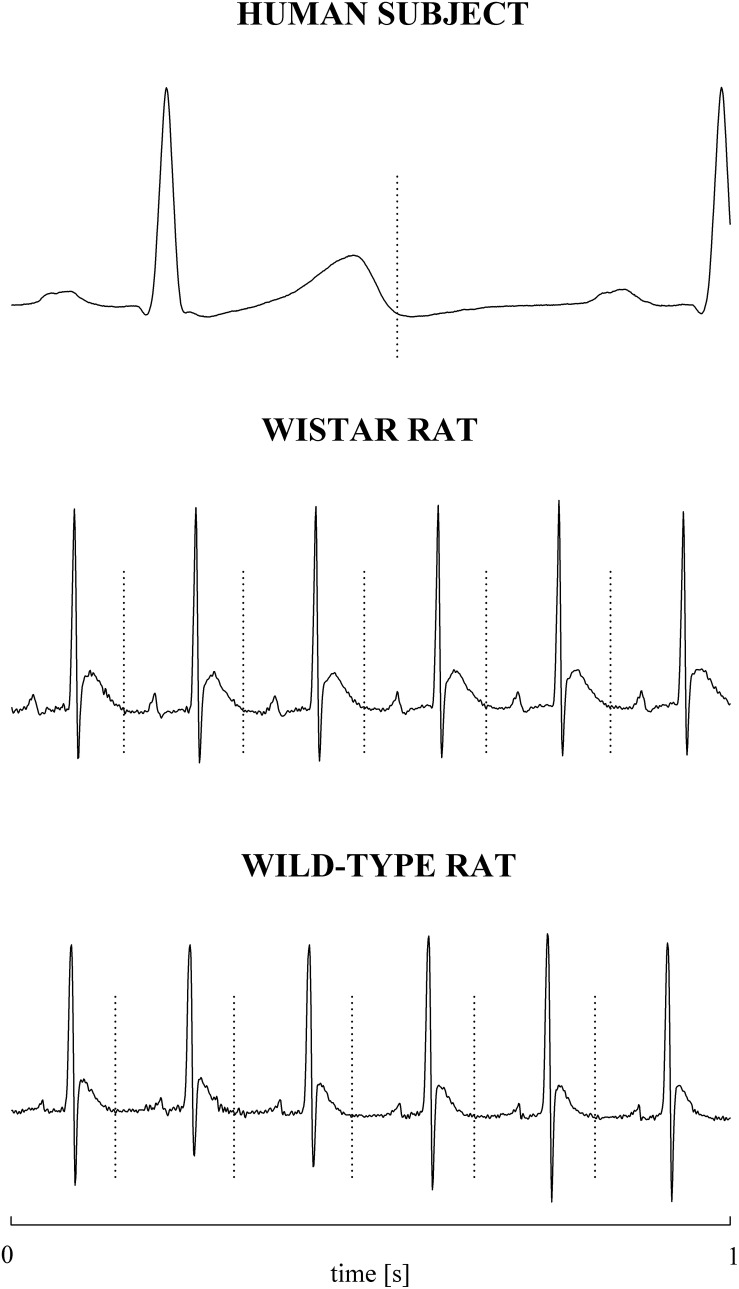
Examples of T-wave end delineation in ECG signals of a healthy young human subject **(top)**, WI rat **(middle)**, and WT rat **(bottom)** in basal condition. T-wave end is marked with a vertical dotted line.

**FIGURE 2:**
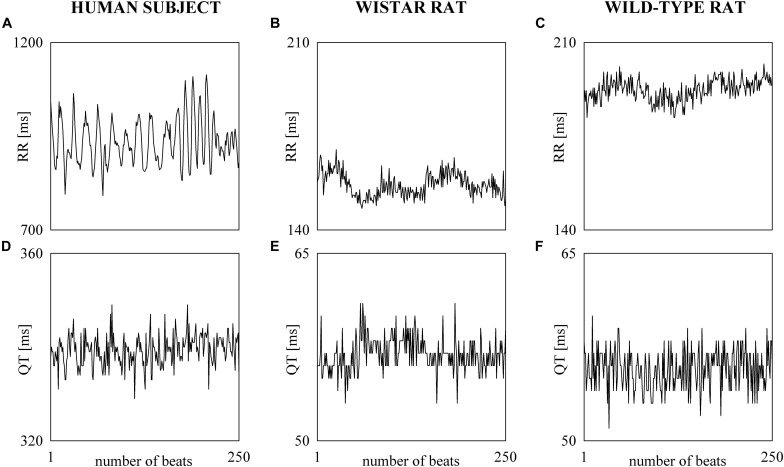
The line plots show examples of beat-to-beat RRV **(A–C)** and QTV **(D–F)** series undergoing power spectral analysis recorded in a healthy young human subject **(A,D)**, WI rat **(B,E)**, and WT rat **(C,F)**.

The electrocardiographic traces recorded in both healthy humans and rats were processed with a software, developed in house, automatically measuring RR and QT (Porta et al., 1998b). The peak of the QRS complex (i.e., the R-wave) was automatically located via a method based on a threshold on the first derivative of the electrocardiogram. The peak of the QRS complex was fixed via parabolic interpolation. The RR was measured as the time distance between two consecutive QRS complex peaks. The QT was approximated as the time interval between the peak of the QRS complex and the T-wave offset (RTend) (Porta et al., 2010, 2011). The end of the T-wave was automatically delineated where the absolute value of the first derivative calculated on T-wave downslope became smaller than 30% of the absolute value of the steepest slope of the T-wave. Figure 1 shows an example of the automatic detection of the T-wave end in a healthy subject (top panel), a WI rat (middle panel), and a WT rat (bottom panel). The detections of the QRS complex were visually checked and corrected in case of identification errors and in this case the T-wave offset delineation procedure was run again starting from the new position of the QRS complex. T-wave end detections were checked to assure the quality of the T-wave delineation. Problematic T-wave morphologies such as biphasic shapes were not observed and the first return to the isoelectric line after the onset of the T-wave always denotes the offset of the repolarization period in both humans and rats. The effects of isolated ectopic beats on RR and QT beat-to-beat series were corrected by means of cubic spline interpolation starting from the RR and QT measures unaffected by non-sinus cardiac beats. Corrections never exceeded the 5% of the total beats. Within each experimental session of the human protocol (i.e., REST, T45, R45, T90, and R90) segments of 250 consecutive RR and QT measures were selected. Stationarity of the selected sequences was tested according to Magagnin et al. (2011). The first stationary sequence found 3 min after the onset of posture changes was taken as the representative segment during T45 and T90 sessions and the first stationary sequence 10 min after returning to the supine position after head-up tilt was taken as the representative segment during R45 and R90 sessions. As to the animal protocol, a 10-min segment was selected in a random position within the overall recording session. The analysis was carried out over the 10-min segments divided into adjacent windows of 250 consecutive RR and QT measures with 80% overlap. The median of the distribution was chosen as the representative value of the whole series. Figure 2 shows some examples of beat-to-beat RRV and QTV series derived from a human subject at REST (Figures 2A,D), from a WI rat (Figures 2B,E) and a WT rat (Figures 2C,F) in unstressed conditions.

### Time and Frequency Domain RRV and QTV Analyses

In the time domain, we computed the mean of RR and QT beat-to-beat series (μ_RR_ and μ_QT_, respectively). μ_RR_ and μ_QT_ were expressed in ms. Linear detrending procedure, subtracting from the original series the best fit linear trend, was exploited to prevent the drift of the mean and favor stationarity. After linear detrending of the series, the variances of RR and QT beat-to-beat series ($\sigma_{\text{RR}}^{2}$ and $\sigma_{\text{QT}}^{2}$, respectively) were calculated and expressed in ms^2^. Parametric power spectral analysis was performed. RRV and QTV series were modeled as realizations of an autoregressive process. The coefficients of the autoregressive process and the variance of the white noise corrupting the determinist part of the process were estimated via least squares method solved via the Levinson–Durbin recursion (Kay and Marple, 1981). The number of coefficients was optimized via Akaike’s (1974) criterion within the range from 10 to 16. Power spectral density was decomposed into power spectral components (Baselli et al., 1997), classified as LF or HF component, according to their central frequency. The LF band range was 0.04–0.15 Hz for humans (Task Force of the European Society of Cardiology and the North American Society of Pacing and Electrophysiology,, 1996) and 0.2–0.75 Hz for rats (Carnevali et al., 2013), while the HF band range was 0.15–0.5 Hz for humans (Task Force of the European Society of Cardiology and the North American Society of Pacing and Electrophysiology,, 1996) and 0.75–2.5 Hz for rats (Carnevali et al., 2013). The sum of the absolute power of all HF components of the RR series was termed as HF_RR_ and considered to be an index of vagal modulation directed to the sinus node (Pomeranz et al., 1985), whereas the sum of the absolute power of all LF components of the QT series was labeled LF_QT_ and considered to be an index of sympathetic modulation directed to the heart (Porta et al., 2010, 2011; Baumert et al., 2011; El-Hamad et al., 2015). The power of RRV in the LF band, indicated as LF_RR_, and the power of the QTV in the HF band, labeled as HF_QT_, were computed as well. LF_RR_, HF_RR_, LF_QT_, and HF_QT_ indexes were given in absolute units and expressed in ms^2^. Spectral analysis was carried out over the linearly detrended RRV and QTV series.

### Statistical Analysis

In the human protocol one-way repeated measures analysis of variance (Dunnett’s test for multiple comparisons) was performed to check the significance of the differences of T45, R45, T90, and R90 versus REST. If the Kolmogorov–Smirnov normality test was not passed, Friedman one-way repeated measures analysis of variance on ranks (Dunnett’s test for multiple comparisons) was carried out. In the animal protocol unpaired *t*-test was performed to assess the significance of the differences between the strains (WI versus WT) and subgroups (AGG versus non-AGG). If the Kolmogorov–Smirnov normality test was not passed, Mann–Whitney rank sum test was carried out. Statistical analysis was carried out using a commercial statistical program (Sigmaplot, Systat Software, Inc., Chicago, IL, United States, version 11.0). A *p* < 0.05 was always considered as significant.

## Results

**FIGURE 3:**
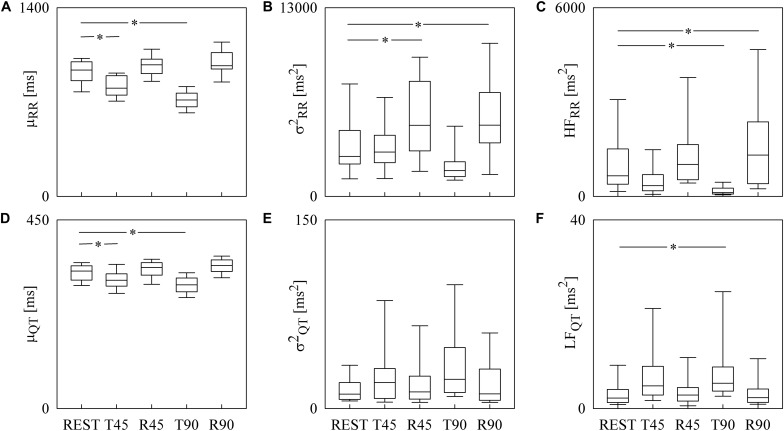
The box-and-whisker graphs show the results of the time and frequency domain analyses of RRV and QTV in healthy young humans. The μ_RR_ **(A)**, $\sigma_{\text{RR}}^{2}$ **(B)**, HF_RR_ **(C)**, μ_*Q*__*T*_ **(D)**, $\sigma_{\text{QT}}^{2}$ **(E)**, and LF_QT_ **(F)** are given as a function of the experimental condition (i.e., REST, T45, R45, T90, and R90). Box height represents the interquartile range, median is marked with a horizontal solid segment, and whiskers denote the 10th and 90th percentile. The symbol ^∗^ indicates a *p* < 0.05 versus REST.

Box-and-whisker plots of Figure 3 show the results of RRV (Figures 3A–C) and QTV (Figures 3D–F) analyses performed on human data as a function of the experimental condition (i.e., REST, T45, R45, T90, and R90). Compared to REST, μ_RR_ decreased during both T45 and T90, while it was unchanged during R45 and R90 (Figure 3A). $\sigma_{\text{RR}}^{2}$ was significantly higher during both R45 and R90 and was not affected by the orthostatic challenge (Figure 3B). HF_RR_ power significantly decreased during T90 and increased during R90 compared to REST (Figure 3C). μ_QT_ was significantly reduced during both T45 and T90 and did not vary during R45 and R90 (Figure 3D). $\sigma_{\text{QT}}^{2}$ did not change with the experimental condition (Figure 3E). LF_QT_ power increased during T90 and remained stable in all the other experimental conditions (Figure 3F). Results relevant to time and frequency domain RRV and QTV markers derived from the experimental protocol on humans are summarized in Table 1. The same table reports the LF_RR_ and HF_QT_ powers as well. Both these latter markers did not vary with the experimental condition.

**TABLE 1:** Results of the time and frequency domain analyses of RRV and QTV in healthy young subjects.

**Index**	**REST**	**T45**	**R45**	**T90**	**R90**
μ_RR_ (ms)	937.18 (135.11)	802.56 (142.08)*	975.94 (97.25)	715.18 (96.47)*	969.17 (117.36)
$\sigma_{\text{RR}}^{2}$ (ms^2^)	2755.2 (2083.51)	3049.86 (1596.87)	4894.99 (4585.21)*	1773.36 (940.24)	4904.22 (3447.03)*
LF_RR_ (ms^2^)	952.56 (1219.09)	1180.74 (1161.83)	1694.45 (1840.67)	890.56 (766.05)	1372.49 (1741.51)
HF_RR_ (ms^2^)	651.6 (1095.69)	342.08 (479.25)	1016.27 (1040.69)	117.27 (182.51)*	1312.98 (1651.28)*
μ_QT_ (ms)	327.91 (33.6)	306.05 (26.65)*	336.25 (27.46)	295.09 (30.19)*	341.03 (26.73)
$\sigma_{\text{QT}}^{2}$ (ms^2^)	11.43 (12.29)	20.67 (23.11)	13.13 (16.72)	23.21 (33.49)	11.59 (23.18)
LF_QT_ (ms^2^)	2.2 (3.98)	4.8 (5.71)	2.86 (2.76)	5.36 (4.78)*	2.29 (2.5)
HF_QT_ (ms^2^)	4.45 (5.91)	7.43 (12.09)	3.34 (9.89)	8.62 (11.9)	4.09 (11.28)

*REST, supine position; T45, head-up tilt at 45°; R45, recovery in supine position after T45; T90, head-up tilt at 90°; R90, recovery in supine position after T90; LF, low frequency; HF, high frequency; RR, time interval between two consecutive QRS complexes; QT, time interval between the peak of the QRS complex and the T-wave offset; RRV, RR variability; QTV, QT variability; LF_*RR*_, RRV power in the LF band expressed in absolute units; HF_RR_, RRV power in the HF band expressed in absolute units; LF_*QT*_, QTV power in the LF band expressed in absolute units; HF_*QT*_, QTV power in the HF band expressed in absolute units. Results are presented as median with the interquartile range in round brackets. The symbol ^∗^ indicates a *p* < 0.05 versus REST.*

**FIGURE 4:**
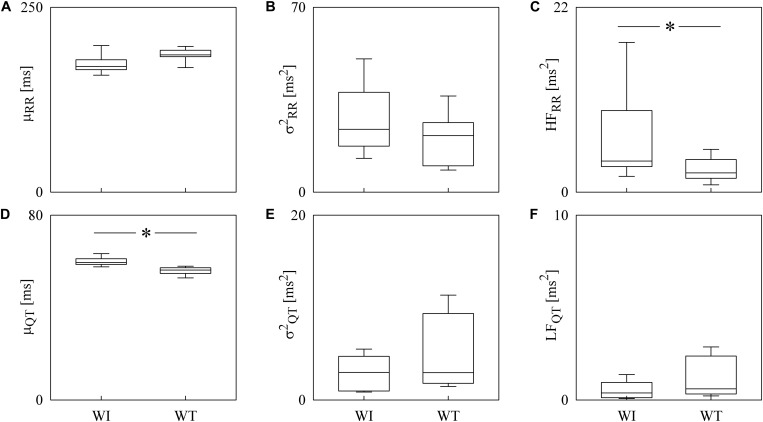
The box-and-whisker graphs show the results of the time and frequency domain analyses of RRV and QTV in rats. The μ_RR_ **(A)**, $\sigma_{\text{RR}}^{2}$ **(B)**, HF_RR_ **(C)**, μ_QT_ **(D)**, $\sigma_{\text{QT}}^{2}$ **(E)**, and LF_QT_ **(F)** are given as a function of the strain (i.e., WI and WT). Box height represents the interquartile range, median is marked with a horizontal solid segment, and whiskers denote the 10th and 90th percentile. The symbol ^∗^ indicates a *p* < 0.05 versus WI rats.

**FIGURE 5:**
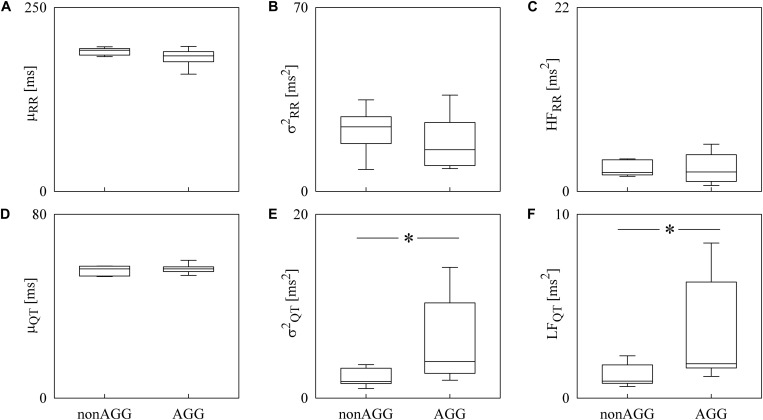
The box-and-whisker graphs show the results of the time and frequency domain analyses of RRV and QTV in WT rats classified into non-AGG and AGG animals. The μ_RR_ **(A)**, $\sigma_{\text{RR}}^{2}$ **(B)**, HF_RR_ **(C)**, μ_QT_ **(D)**, $\sigma_{\text{QT}}^{2}$ **(E)**, and LF_QT_ **(F)** are given as a function of the WT subcategory (i.e., non-AGG and AGG). Box height represents the interquartile range, median is marked with a horizontal solid segment, and whiskers denote the 10th and 90th percentile. The symbol ^∗^ indicates a *p* < 0.05 versus non-AGG animals.

Box-and-whisker plots of Figure 4 show the results of RRV (Figures 4A–C) and QTV (Figures 4D–F) analyses performed on data derived from WI and WT rats. μ_RR_ (Figure 4A) and $\sigma_{\text{RR}}^{2}$ (Figure 4B) were similar between the two strains, while the HF_RR_ power (Figure 4C) was higher in WI compared to WT rats. μ_QT_ (Figure 4D) was longer in WI rats, while no strain differences in $\sigma_{\text{QT}}^{2}$ (Figure 4E) and LF_*Q*__*T*_ power (Figure 4F) were observed. Figure 5 has the same structure as Figure 4 but it shows the results of RRV (Figures 5A–C) and QTV (Figures 5D–F) analyses performed on data obtained from WT rats that were classified as non-AGG and AGG. μ_RR_ (Figure 5A), $\sigma_{\text{RR}}^{2}$ (Figure 5B), HF_RR_ (Figure 5C), and μ_*Q*__*T*_ (Figure 5D) did not differentiate the two subgroups. On the contrary, $\sigma_{\text{QT}}^{2}$ (Figure 5E) and LF_QT_ (Figure 5F) were able to separate the two groups of WT rats, being both $\sigma_{\text{QT}}^{2}$ and LF_QT_ power higher in AGG than in non-AGG animals. RRV and QTV markers derived from the experimental protocol on rats are summarized in Tables 2, 3. These tables reported LF_RR_ and HF_QT_ powers as well. Both these markers were similar in WI and WT animals (Table 2) and they were not able to distinguish non-AGG from AGG animals (Table 3).

**TABLE 2:** Results of time and frequency domain analyses of RRV and QTV in WI and WT rats.

**Index**	**WI**	**WT**
μ_RR_ (ms)	169.99 (7.37)	185.6 (8.31)
$\sigma_{\text{RR}}^{2}$ (ms^2^)	23.76 (19.62)	21.38 (15.5)
LF_RR_ (ms^2^)	1.22 (1.4)	0.58 (0.37)
HF_RR_ (ms^2^)	3.69 (4.63)	2.29 (2.16)*
μ_QT_ (ms)	59.54 (2.21)	56.23 (2.32)*
$\sigma_{\text{QT}}^{2}$ (ms^2^)	2.98 (3.49)	2.95 (6.01)
LF_QT_ (ms^2^)	0.38 (0.78)	0.6 (1.63)
HF_QT_ (ms^2^)	1.36 (1.93)	1.68 (3.76)

*WI, Wistar rats; WT, wild-type Groningen rats; LF, low frequency; HF, high frequency; RR, time interval between two consecutive QRS complexes; QT, time interval between the peak of the QRS complex and the T-wave offset; RRV, RR variability; QTV, QT variability; LF_*RR*_, RRV power in the LF band expressed in absolute units; HF_*RR*_, RRV power in the HF band expressed in absolute units; LF_*QT*_, QTV power in the LF band expressed in absolute units; HF_*QT*_, QTV power in the HF band expressed in absolute units. Results are presented as median with the interquartile range in round brackets. The symbol ^∗^ indicates a *p* < 0.05 versus WI.*

**TABLE 3:** Results of time and frequency domain analyses of RRV and QTV in WT rats classified as non-AGG and AGG animals.

**Index**	**non-AGG**	**AGG**
μ_RR_ (ms)	191.9 (7.27)	184.22 (11.4)
$\sigma_{\text{RR}}^{2}$ (ms^2^)	24.57 (4.83)	15.86 (12.84)
LF_RR_ (ms^2^)	0.66 (0.24)	0.53 (0.45)
HF_RR_ (ms^2^)	2.26 (1.71)	2.32 (2.89)
μ_QT_ (ms)	56.21 (4.2)	56.25 (1.9)
$\sigma_{\text{QT}}^{2}$ (ms^2^)	1.8 (1.37)	3.98 (7.5)*
LF_QT_ (ms^2^)	0.3 (0.54)	0.67 (1.96)*
HF_QT_ (ms^2^)	0.92 (0.78)	1.87 (4.59)

*WT, wild-type Groningen rats; non-AGG, non-aggressive WT rats; AGG, aggressive WT rats; LF, low frequency; HF, high frequency; RR, time interval between two consecutive QRS complexes; QT, time interval between the peak of the QRS complex and the T-wave offset; RRV, RR variability; QTV, QT variability; LF_*RR*_, RRV power in the LF band expressed in absolute units; HF_*RR*_, RRV power in the HF band expressed in absolute units; LF_*QT*_, QTV power in the LF band expressed in absolute units; HF_*QT*_, QTV power in the HF band expressed in absolute units. Results are presented as median with the interquartile range in round brackets. The symbol ^∗^ indicates a *p* < 0.05 versus non-AGG.*

## Discussion

To the best of our knowledge this is the first study in which QTV parameters were evaluated in rats concomitantly with traditional RRV measures for the assessment of cardiac autonomic control and a parallel between human and rat RRV and QTV markers was drawn. The most important findings of this study can be summarized as follows: (i) RRV is descriptive of the cardiac vagal regulation in both humans and rats; (ii) QTV is representative of cardiac sympathetic control in both humans and rats; (iii) results of RRV and QTV should be simultaneously considered to more deeply describe cardiac autonomic control in both humans and rats.

### RRV and QTV Provide Complementary Information About Cardiac Autonomic Control in Humans and Rats

One of the major difficulties in exploiting RRV and spectral markers derived from RRV analysis to comprehensively characterize cardiac autonomic control is the strong link of RRV with the variation of vagal autonomic outflow, while its sensitivity to changes of the activity of the sympathetic autonomic limb is more limited. Indeed, since the initial studies on RRV (Akselrod et al., 1981; Pomeranz et al., 1985) it is well-known that the HF_RR_ power is completely abolished by full vagal blockade carried out via a high dose of atropine and that the same pharmacological challenge affects remarkably the LF_RR_ power as well. This observation suggested that the HF_RR_ power is a genuine marker of vagal modulation directed to the sinus node, while the LF_RR_ power results from the changes of the activity of both sympathetic and vagal limbs of the autonomic nervous system (Akselrod et al., 1981; Pomeranz et al., 1985). Normalization strategies attempted to limit the dependence of the LF_RR_ power on cardiac vagal control. For example, the ratio of the LF_RR_ power to $\sigma_{\text{RR}}^{2}$ minus the RRV power in the very LF band, known as LF_RR_ power expressed in normalized units (Pagani et al., 1986), is one of the most frequently exploited normalized RRV indexes. The attempts of normalizing frequency domain markers of RRV to achieve a more genuine marker of sympathetic control generated some controversies (Eckberg, 1997; Pagani et al., 1997; Billman, 2013; Reyes del Paso et al., 2013). Among the most controversial issues there is the non-zero value of normalized LF_RR_ power after full vagal blockade in presence of null RR changes and the strict link between normalized LF_RR_ and normalized HF_RR_ powers given that their sum is 100 (Eckberg, 1997). The final result is that no normalization procedure solved the original problem due to the inherent contribution of vagal limb to RRV in the LF band (Akselrod et al., 1981; Pomeranz et al., 1985). More recently, some studies on QTV have suggested the possibility of monitoring cardiac sympathetic control via markers extracted from QTV (Porta et al., 1998a, 2010; Berger, 2009; Malik, 2009; El-Hamad et al., 2015; Baumert et al., 2016) and have outlined the clinical relevance of this approach in pathological populations and risk stratification (Berger et al., 1997; Atiga et al., 1998; Baumert et al., 2008, 2011; Bari et al., 2014; Porta et al., 2015). A pragmatic route to face the issue generated by the debate on the use of RRV markers in the frequency domain was made operational in Porta et al. (2015) who proposed the simultaneous exploitation of RRV and QTV to derive a frequency domain description of the cardiac vagal control via the HF_RR_ power and of the cardiac sympathetic control via the LF_QT_ power. The strategy proposed in Porta et al. (2015) was tested in this study in humans during an experimental protocol evoking sympathetic activation and vagal withdrawal (i.e., head-up tilt) and the progressive sympathetic regulation departure and vagal control rebound during recovery after the postural challenge (Montano et al., 1994; Cooke et al., 1999; Porta et al., 2011; Marchi et al., 2016) and in rats featuring documented differences in cardiac sympatho-vagal balance at baseline (Carnevali et al., 2013; Carnevali and Sgoifo, 2014). The present study outlines the ability of the simultaneous exploitation of the HF_RR_ and LF_QT_ markers in typifying state- and trait-related modifications of the cardiac autonomic regulation in human and animal experiments. In the human protocol the significant decrease of the HF_RR_ marker during T90 and the concomitant increase of LF_QT_ power suggest, respectively, a reduced vagal and an augmented sympathetic controls as it is expected in response to the postural challenge (Montano et al., 1994; Cooke et al., 1999; Porta et al., 2011; Marchi et al., 2016). The specific ability of the HF_RR_ marker in tracking the cardiac vagal control was emphasized by the particular design of the experimental protocol in humans considering the period of recovery after the postural challenge. Indeed, the greater cardiac vagal regulation regaining after T90 was stressed by the increase of the HF_RR_ power above the levels observed at REST. The independence of the LF_QT_ power from the level of cardiac vagal control was supported by the stable values of this index during recovery compared to REST, thus stressing the complementary information that can be derived from the joint use of HF_RR_ and LF_QT_ markers. The strategy proposing the concomitant use of HF_RR_ and LF_QT_ powers excludes the utilization of the LF_RR_ power due to its mixed nature and that of the HF_QT_ power due to its non-autonomic origin. The mixed origin of the LF_RR_ power is supported by the present study as well: indeed, the constancy of the LF_RR_ power as a function of the experimental condition in the head-up tilt protocol and the inability of the LF_RR_ power to distinguish non-AGG from AGG rats is in agreement with a simultaneous increase of sympathetic modulation and a decrease of the vagal one (Porta et al., 2011). The non-autonomic origin of the HF_QT_ power results from the observation that it is likely to be the consequence of the projection of cardiac axis movements due to respiration over a single lead given that it increased when assessed over Z lead compared to X and Y ones (Porta et al., 1998b) and it is present in subjects under cardiac pacing (Lombardi et al., 1996). The non-autonomic nature of the HF_QT_ power was supported by the present study as well: indeed, it is invariable in both human and animal protocols.

The proposed strategy has the inherent limitation of disregarding the dependence of QTV on RRV due to the well-known relation linking QT to the preceding RR (Bazett, 1929). However, the selection of spectral indexes computed in different frequency bands (i.e., HF_RR_ and LF_QT_ powers) should mitigate the effects of this dependence. Our result corroborates this observation given that in humans during R90 the HF_RR_ power increased compared to REST, while the LF_QT_ marker remained stable, and in rats only the LF_QT_ power was greater in the AGG group compared to the non-AGG one while the HF_RR_ power was unvaried. However, models of the dynamical dependence of QTV on RRV should be tested (Porta et al., 1998a, 2010) in future to understand whether some normalization procedure should be applied to better represent the genuine contribution of the sympathetic drive directed to the ventricles.

### RRV and QTV Can Be Fruitfully Exploited for Cardiac Autonomic Characterization in Rats

To the best of our knowledge, this is the first study in which QTV analysis was carried out on rats with the aim at assessing cardiac autonomic control and QTV markers were discussed along with those derived from RRV analysis. This approach was successfully applied with the aim at differentiating WI and WT rats and divergent subpopulations within the WT strain. WI rats are highly domesticated, docile, and placid, while WT rats exhibit a more aggressive behavior during a social conflict (Buwalda et al., 2011) than WI rats. These differences in trait aggressiveness between the two strains are mirrored by a different state of the sympatho-vagal balance in unstressed conditions, with WT rats generally showing lower indexes of cardiac vagal modulation than WI counterparts (Carnevali and Sgoifo, 2014). Our results are in agreement with Carnevali and Sgoifo (2014) given that we found a lower HF_RR_ power in WT rats than in WI rats. The expected increase of the LF_QT_ marker, suggesting a higher sympathetic control in WT rats than in WI animals, was not found even though a tendency toward an increase of the LF_QT_ power was evident. Since in presence of an active sympatho-vagal balance it is expected that a significant increase of HF_RR_ power is associated to a significant decrease of the LF_QT_ one, the decrease of HF_RR_ power in WT animals in association with an unvaried LF_QT_ index might suggest a greater complexity of the interactions between vagal and sympathetic branches of the autonomic nervous system. Complex interactions between the two branches of the autonomic nervous system are known to lead to imbalanced situations in which a vagal withdrawal is not linked to a simultaneous and proportional sympathetic activation or *vice versa* (Porta et al., 2007) or situations featuring co-activation or co-inhibition of both the autonomic nervous system limbs (Kollai and Koizumi, 1979; Paton et al., 2005). These situations might lead to non-reciprocal trends in cardiac vagal and sympathetic controls (Kollai and Koizumi, 1979). The complexity of the sympatho-vagal interactions requires a more flexible tool that does not pretend to quantify cardiac autonomic control from a unique variability series like RRV-based analysis, but considers the joint observation of RRV and QTV as a mandatory standpoint for the reliable inference of autonomic nervous system state.

The relevance of the simultaneous assessment of RRV and QTV is even more evident when the WT rats were subdivided into non-AGG and AGG animals (de Boer et al., 2003). In previous studies, AGG rats were found to be characterized by lower RRV markers in unstressed conditions compared to non-AGG rats, thus suggesting that the aggressive behavior is associated with a lower vagal control (Carnevali et al., 2013; Carnevali and Sgoifo, 2014). Such a low cardiac vagal modulation was associated with a higher arrhythmia susceptibility and a greater vulnerability to cardiac morbidity in the AGG group (Carnevali et al., 2013). Differences in resting autonomic modulation between AGG and non-AGG rats were not evident in the current study using the RRV markers given that the HF_RR_ power was similar, but they were unveiled by the QTV markers given that the LF_QT_ power was greater in AGG than in non-AGG rats. Therefore, our results suggest that the AGG rats are characterized by a higher resting sympathetic modulation that is not accompanied by a concomitant reduction of vagal modulation. This finding might be another evidence of the complexity of the cardiac control in rats where a high sympathetic drive does not imply by necessity a vagal withdrawal and further corroborates the need of an approach to the study of the cardiac autonomic control integrating different signals and not necessarily based on the concept of sympatho-vagal balance.

### Time Domain RRV and QTV Parameters Versus Spectral RRV and QTV Markers

Time domain markers were commonly shown to provide the representation of the effect of a physiological challenge or an experimental maneuver on the cardiovascular system. For example, in our human protocol, the trend of the μ_RR_ suggests that the orthostatic challenge was effective because the reduction of the venous return due to posture modification provokes a tachycardic response in the attempt to prevent the arterial pressure drop (Montano et al., 1994; Cooke et al., 1999; Porta et al., 2011; Marchi et al., 2016). For example, in the same protocol the evolution of μ_QT_ suggests that the QT measures are reliable given that it is well-known that in humans μ_QT_ is shorter when μ_RR_ is reduced (Bazett, 1929). However, the limits of time domain measures in providing a complete picture appear evidently as well. For example, $\sigma_{\text{QT}}^{2}$ was less powerful than the LF_QT_ power in describing the effect of the orthostatic challenge likely because non-autonomic effects resulting from cardiac axis movements synchronous with respiration (Porta et al., 1998b) are likely to influence more remarkably $\sigma_{\text{QT}}^{2}$ than its portion in the LF band. For example, in non-AGG and AGG rats the μ_RR_ and μ_QT_ were similar, while the LF_QT_ power increased in the AGG group, thus stressing the non-redundant nature of time and frequency domain markers.

### On the Use of Rats as an Animal Model of Human Cardiac Autonomic Control Explored via RRV and QTV Analyses

Rats are considered animals exhibiting a sympathetic dominance given that their intrinsic heart rate (i.e., the cardiac frequency under complete pharmacological autonomic blockade) is lower than the resting heart rate (Opthof, 2000). However, this observation does not imply that vagal control is absent. Indeed, the full muscarinic receptor blockade induced via a high dose of atropine dramatically reduced RRV (Japundzic et al., 1990; Cerutti et al., 1991; Silva et al., 2017), thus supporting the observation that changes of vagal activity contribute importantly to $\sigma_{\text{RR}}^{2}$ and corroborating the use of these animals in translational studies on cardiac autonomic control. More importantly for the present study, rats respond differently to sympathetic stimulation: indeed, they show a QT prolongation, while in humans a QT shortening is observed (Conrath and Opthof, 2006; Speerschneider and Thomsen, 2013). The parallel changes of μ_RR_ and μ_QT_ reported in the present study in the human protocol and the opposite trends of μ_RR_ and μ_QT_ in WI and WT groups are in agreement with the diverse effect of an augmented sympathetic drive on μ_RR_ and μ_QT_ in humans and rats. In spite of this peculiarity, the RRV and QTV markers seem to maintain similar interpretation in both species. However, the lack of application of a stressor inducing a sympathetic activation in both WI and WT rats prevents us to deepen this issue.

### Limitations of the Study and Future Developments

While our data support the association between QTV magnitude and sympathetic control, they are less informative about the shape of the relation between them. It is likely that the QTV could reflect mean sympathetic activity and its modifications about the mean when sympathetic drive is sufficiently high, while below a certain mean neural activity value QTV could be useless. We advocate pharmacological studies that could graduate the challenge in a finer manner and the contemporaneous direct recording of sympathetic activity to provide insight on the shape of this relation.

There is an open debate on the dependency of the magnitude of RRV and QTV on their means and on the need of some normalization (Sacha and Pluta, 2008; Sacha, 2013; Boyett et al., 2019; Malik et al., 2019). In the present study we tested the redundancy between QTV and μ_QT_ by calculating the normalized QT variance (QTVN), namely the ratio of the square QT standard deviation to the square μ_QT_ (Baumert et al., 2016). No difference was found either among experimental conditions in the human protocol or between groups in the animal protocol. This result might suggest a certain degree of dependency between QTV and μ_QT_. However, the lack of significant differences is due to the enormous standard deviation of QTVN, sometimes close to two times the QTVN mean. This observation suggests some caution in using QTVN given that normalization procedure might behave differently at diverse values of μ_QT_ and the need of more deeply exploring the relation between QTV and μ_QT_.

Since in rats the T-wave morphology is different from that in humans, due to the different shapes of the ventricular action potentials (Fabritz et al., 2010; Boukens et al., 2014), future studies should be focused on the comparison of methods based on a threshold on the first derivative (Laguna et al., 1990; Nollo et al., 1992; Porta et al., 1998b), on the tangent method taking the interception between the straight line at the steepest point of the T-wave downslope and the isoelectric line (Lepeschkin and Surawicz, 1952; Yamada et al., 1993; Porta et al., 1998b) and on template matching approach (Berger et al., 1997; Baumert et al., 2012).

## Conclusion

In the present study, we computed frequency domain markers concurrently derived from RRV and QTV for a deeper characterization of the cardiac autonomic control. The power of RRV in the HF band and the power of QTV in the LF band were exploited to typify state- and trait-related modifications of the cardiac autonomic regulation in humans and rats. We found that the information derived from RRV and QTV spectral markers is not redundant given that trends of the HF power of RRV cannot be inferred from those of the LF power of QTV and vice versa. The complementary information was interpreted in relation to the inherent ability of RRV and QTV spectral markers to describe, respectively, cardiac vagal and sympathetic controls. Therefore, we conclude that the concomitant evaluation of RRV and QTV frequency domain markers can provide a more insightful view on cardiac autonomic function in both humans and rats than the sole exploitation of RRV indexes.

## Data Availability Statement

The datasets generated for this study are available on request to the corresponding author.

## Ethics Statement

The studies involving human participants were reviewed and approved by the Human Research and Ethical Review Board of the L. Sacco Hospital, Milan, Italy. The patients/participants provided their written informed consent to participate in this study. The animal study was reviewed and approved by the Veterinarian Animal Care and Use Committee of the University of Parma, Parma, Italy.

## Author Contributions

AP conceived and designed the study. AS, LC, AMT, and AC performed the experiments. BM analyzed the data. BM and AP drafted the manuscript and prepared the figures. BM, VB, AS, LC, BC, EV, AMT, AC, LDV, and AP interpreted the results, edited and revised the manuscript, and approved the final version of the manuscript.

## Conflict of Interest

The authors declare that the research was conducted in the absence of any commercial or financial relationships that could be construed as a potential conflict of interest.
